# Cross-sectional study of 168 patients with hepatorenal tyrosinaemia and implications for clinical practice

**DOI:** 10.1186/s13023-014-0107-7

**Published:** 2014-08-01

**Authors:** Sebene Mayorandan, Uta Meyer, Gülden Gokcay, Nuria Garcia Segarra, Hélène Ogier de Baulny, Francjan van Spronsen, Jiri Zeman, Corinne de Laet, Ute Spiekerkoetter, Eva Thimm, Arianna Maiorana, Carlo Dionisi-Vici, Dorothea Moeslinger, Michaela Brunner-Krainz, Amelie Sophia Lotz-Havla, José Angel Cocho de Juan, Maria Luz Couce Pico, René Santer, Sabine Scholl-Bürgi, Hanna Mandel, Yngve Thomas Bliksrud, Peter Freisinger, Luis Jose Aldamiz-Echevarria, Michel Hochuli, Matthias Gautschi, Jessica Endig, Jens Jordan, Patrick McKiernan, Stefanie Ernst, Susanne Morlot, Arndt Vogel, Johannes Sander, Anibh Martin Das

**Affiliations:** 1 Clinic for Paediatric Kidney-, Liver and Metabolic Diseases, Hannover Medical School, Carl-Neuberg-Str.1, Hannover, D-30625, Germany; 2 Istanbul University Faculty of Medicine, Fatih/Capa, Istanbul 34093, Turkey; 3 Reference Center for Inherited Metabolic Diseases, Hôpital Robert Debré, APHP 48 Boulevard Sérurier, Paris, F-75019, France; 4 Section of Metabolic Diseases, Beatrix Children’s Hospital, University Medical Center Groningen, University of Groningen, Hanzeplein 1, Groningen, 9713 GZ, The Netherlands; 5 Department of Pediatrics, First Faculty of Medicine, Charles University, Prague, Ke Karlovu 2, Prague 2 128 08, Czech Republic; 6 Queen Fabiola Children’s University Hospital, Avenue Crocq 15, Brussels, B-1020, Belgium; 7 Allgemeine Kinderheilkunde und Jugendmedizin, Universitätsklinikum Freiburg, Mathildenstr. 1, Freiburg, D-79106, Germany; 8 Universitätsklinikum Düsseldorf, Moorenstr. 5, Düsseldorf, D-40225, Germany; 9 Bambino Gesù Children’s Hospital, Piazza Sant’Onofrio, 4, Rome, 00165, Italy; 10 Universitätsklinik für Kinder- und Jugendheilkunde, Währinger Gürtel 18-20, Wien, 1090, Austria; 11 Medizinische Universität Graz, Auenbruggerplatz 2, Graz, A-8036, Austria; 12 Dr.von Haunersches Kinderspital, Lindwurmstr. 4, München, D-80337, Germany; 13 Unidad de Enfermedades Metabólicas Congénitas, Hospital Clínico Universitario, Santiago de Compostela, Travesía de Choupana, s/n 15706, Santiago de Compostela, Spain; 14 Universitätsklinikum Hamburg-Eppendorf, Klinik und Poliklinik für Kinder-und Jugendmedizin, Martinistr. 52, Hamburg, D-20246, Germany; 15 Department für Kinder-und Jugendheilkunde, Medizinische Universität Innsbruck, Anichstrasse 35, Innsbruck, A-6020, Austria; 16 Pediatric Metabolic Disorders, Rambam Medical center, 6 Ha’Aliya Street, Haifa 31096, Israel; 17 Oslo University Hospital, Nydalen, Oslo, 0424, Norway; 18 Klinik für Kinder-und Jugendmedizin, Steinenbergstr. 31, Reutlingen, D-72764, Germany; 19 Hospital universitario de Cruces, Plaza de Cruces, 12, San Vicente de Barakaldo, 48903, Spain; 20 Universitätspital Zürich, Klinik für Endokrinologie, Diabetologie und Klinische Ernährung, Rämistrasse 100, Zürich, CH-8091, Switzerland; 21 Universitätsklinik für Kinderheilkunde, Inselspital, Freiburgstrasse 7, Bern, CH-3010, Switzerland; 22 Hannover Medical School, Clinic for Gastroenterology, Hepatology and Endocrinology, Carl-Neuberg-Str.1, Hannover, D-30625, Germany; 23 Institute of Clinical Pharmacology, Hannover Medical School, Carl-Neuberg-Str.1, Hannover, D-30625, Germany; 24 Birmingham Children’s Hospital, Steelhouse Ln, Birmingham B4 6NH, United Kingdom; 25 Hannover Medical School, Institute for Biometry, Carl-Neuberg-Str.1, Hannover, D-30625, Germany; 26 Hannover Medical School, Institute for Cellular and Molecular Pathology, Carl-Neuberg-Str.1, Hannover, D-30625, Germany; 27 Screening-Labor Hannover, Am Steinweg 11A/13B, Ronnenberg/Benthe, D-30952, Germany

**Keywords:** Diet, Hepatocellular carcinoma, Liver transplantation, Newborn screening, NTBC, Psychomotor impairment, Succinylacetone, Therapeutic monitoring, Tyrosine

## Abstract

**Background:**

Hepatorenal tyrosinaemia (Tyr 1) is a rare inborn error of tyrosine metabolism. Without treatment, patients are at high risk of developing acute liver failure, renal dysfunction and in the long run hepatocellular carcinoma. The aim of our study was to collect cross-sectional data.

**Methods:**

Via questionnaires we collected retrospective data of 168 patients with Tyr 1 from 21 centres (Europe, Turkey and Israel) about diagnosis, treatment, monitoring and outcome. In a subsequent consensus workshop, we discussed data and clinical implications.

**Results:**

Early treatment by NTBC accompanied by diet is essential to prevent serious complications such as liver failure, hepatocellular carcinoma and renal disease. As patients may remain initially asymptomatic or develop uncharacteristic clinical symptoms in the first months of life newborn mass screening using succinylacetone (SA) as a screening parameter in dried blood is mandatory for early diagnosis. NTBC-treatment has to be combined with natural protein restriction supplemented with essential amino acids. NTBC dosage should be reduced to the minimal dose allowing metabolic control, once daily dosing may be an option in older children and adults in order to increase compliance. Metabolic control is judged by SA (below detection limit) in dried blood or urine, plasma tyrosine (<400 μM) and NTBC-levels in the therapeutic range (20–40 μM). Side effects of NTBC are mild and often transient.

Indications for liver transplantation are hepatocellular carcinoma or failure to respond to NTBC. Follow-up procedures should include liver and kidney function tests, tumor markers and imaging, ophthalmological examination, blood count, psychomotor and intelligence testing as well as therapeutic monitoring (SA, tyrosine, NTBC in blood).

**Conclusion:**

Based on the data from 21 centres treating 168 patients we were able to characterize current practice and clinical experience in Tyr 1. This information could form the basis for clinical practice recommendations, however further prospective data are required to underpin some of the recommendations.

## Background

Hepatorenal tyrosinaemia or tyrosinaemia type 1 (Tyr 1) is a rare autosomal-recessive disorder of tyrosine metabolism with an incidence of 1:125,000 in central Europe. It is more frequent in other regions like Turkey, Quebec and India.

**Figure 1:**
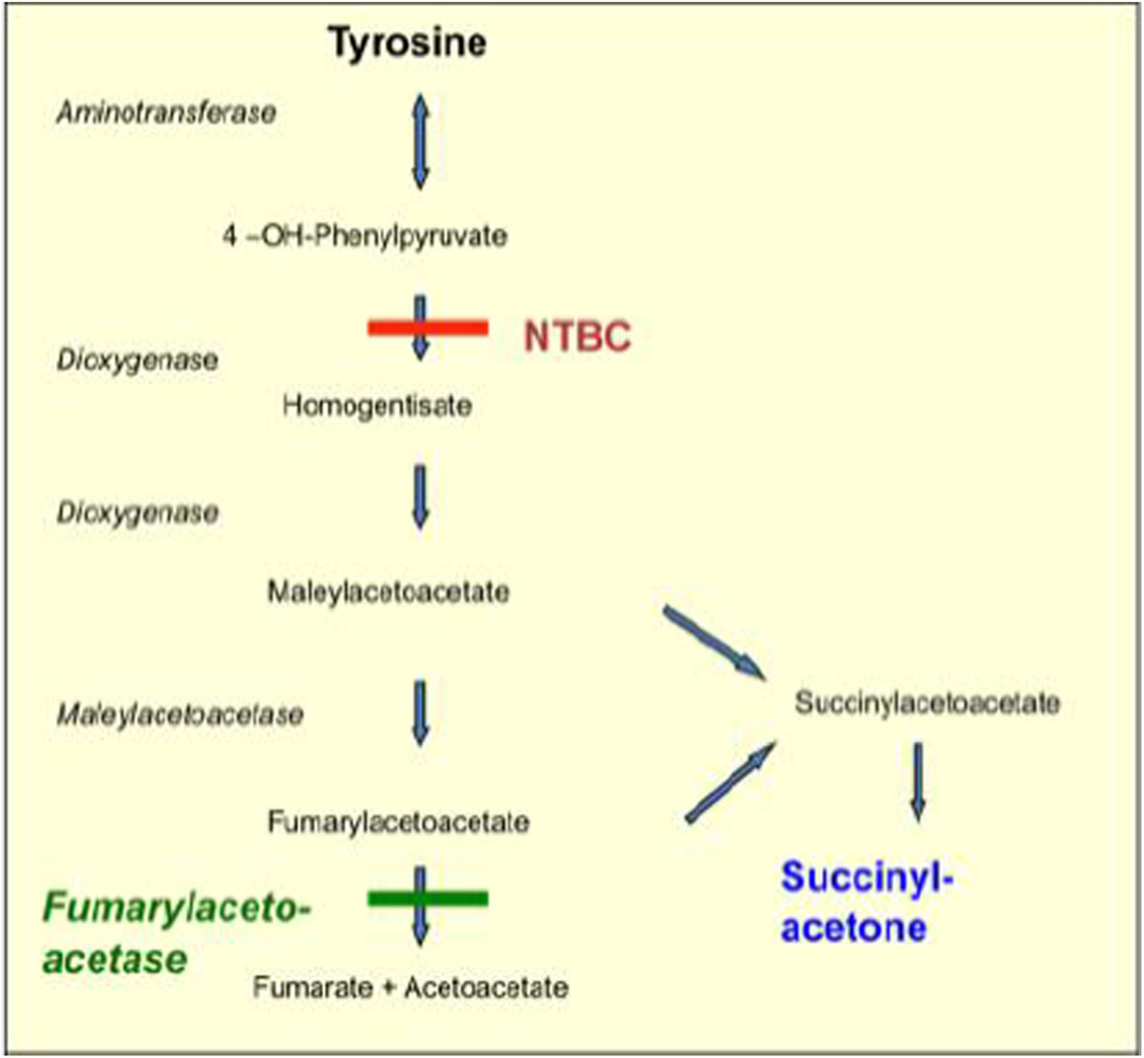
Tyrosine metabolism.

In Tyr 1, the deficiency of the enzyme fumarylacetoacetase causes an accumulation of tyrosine and toxic metabolites (Figure 1) [1]. Tyr 1 mainly affects liver and kidney function, porphyria-like neurological crisis and cardiomyopathy are less frequently observed [2]. Hepatocellular carcinoma is a known long-term complication [3],[4]. Recently, psychomotor impairment has been observed in a number of patients [5]-[8].

Diagnosis is based on the elevated levels of succinylacetone (SA) in urine and/or blood, which is a pathognomonic finding in Tyr 1. Mutation analysis as a diagnostic tool is not routinely performed, except in cases where prenatal diagnosis is planned [9].

The state of the art treatment in Tyr 1 consists of two principles: inhibition of the formation of toxic metabolites by [2-(2-nitro-4-trifluoromethylbenzoyl)-1, 3-cyclohexanedione; nitisinone] NTBC and reduction of tyrosine levels by dietary treatment (Figure 1). SA as a surrogate parameter of toxicity is commonly used for therapeutic monitoring [10].

Despite considerable progress in the diagnosis and management of patients with Tyr 1 in the last 20 years there is lack of a guideline. A review of the literature revealed only few regional studies [11]-[17]. The aim of our study was to collect retrospective cross-sectional data from an international group of physicians treating patients with Tyr 1 in Europe, Turkey and Israel. We sent questionnaires regarding diagnostic procedures, treatment and monitoring of therapy in Tyr 1-patients to metabolic centres treating patients with Tyr 1. Some of the experts later met for a workshop to discuss the findings and possible implications for practical medicine. In order to avoid unintended bias and to stimulate discussion we invited colleagues not directly involved in the clinical management of Tyr 1 (adult hepatologist, geneticist, pharmacologist, screening expert) to attend our expert meeting.

Patients would benefit from a standardized protocol for diagnosis, treatment and monitoring, which would also decrease disparities in health care delivery among different geographical regions and promote reimbursement for drugs, dietary products and therapeutic monitoring by insurance companies.

Recently, recommendations based on personal opinions and experiences from a group of expert clinicians were published [18]. Obviously, randomized multi-centre, placebo-controlled, double blind studies are not possible in inborn errors of metabolism due to their rarity. However, this drawback should not discourage creation of clinical practice recommendations in this group of diseases. As already pointed out by Vockley and coworkers [19],[20] clinical practice recommendations/guidelines may be derived from the integration of evidence-based medicine, review of the literature and consensus from an international expert group.

As only regional studies in Tyr 1 have been published in the past, data from a larger international cohort are urgently required to proceed towards clinical practice recommendations. This prompted us to collect cross-sectional data on diagnosis, management, monitoring and outcome of Tyr 1 in an international cohort.

## Methods

### Workshop participants and data collection

22 metabolic centres from Europe, Turkey and Israel participated in this international survey, 21 centres contributed individual patient data. By this regional limitation we hoped to facilitate the consensus process without falling short of patients.

In preparation for the workshop, we sent questionnaires to the participating centres. Questionnaires consisted of two parts: A general part on basic information and principles of treatment in the single centres and a second part on diagnosis, treatment, monitoring, and outcome in each individual patient. Data from 168 patients could be collected.

Results were presented and discussed during a 2 day workshop in Hannover in November 2012. 21 paediatricians from 12 countries who contributed data participated in the workshop. To assist and stimulate discussions a metabolic dietician (UM), a geneticist (SM), a pharmacologist (JJ), a screening specialist (JS) and 2 hepatologists (AV, JE) specialized in the pathogenesis of HCC complemented the group.

The study was approved by our local ethics review board.

### Outline of the questionnaire

The general part of the questionnaire contained questions on number and age range of patients, staff and frequency of outpatient visits in the single centres.

Centres were asked to name and specify the tools used for the diagnosis of Tyr 1-patients. Furthermore, they were asked to supply data on dietary and medical treatment as well as liver transplantation. This included data on supplementation of vitamins and micronutrients, initial/total daily maintenance dose of NTBC, number of and indication for liver transplantation. In this part of the survey, general information on therapeutic monitoring such as measurement of amino acids, succinylacetone, delta-aminolevulinate and NTBC was collected. Moreover, centres were asked to outline their follow-up procedures such as laboratory tests, imaging, psychomotor/neurological assessment and skeletal as well as ocular examination. The participants had the opportunity to give information on refund of expenses for NTBC, tyrosine-free amino acid mixtures and low-protein food.

The second part of the questionnaire consisted of questions on each individual patient.

This part was divided into:

 demographic data

 diagnosis and clinical presentation

 initial laboratory findings and imaging

 drugs and diet

 NTBC side effects

 monitoring and follow-up

 clinical outcome

### Demographic data

Centres were asked to supply data on the year of birth, sex, current body weight and length, consanguinity, affected family members, ethnic background, schooling (normal or special education) and concomitant diseases.

### Diagnosis and clinical presentation

We asked for the age at diagnosis of each single patient and for specification of diagnostic tools used for newborn screening or selective screening. Participants were asked to itemize initial symptoms and to supply data on mutation analysis of each single patient.

### Initial laboratory findings and imaging

In this part, initial values of AFP/CEA, PT/ PTT, AST/ALT/GGT, plasma phenylalanine/methionine/tyrosine and SA were compiled. Furthermore, centres were asked to specify results of initial imaging.

### Drugs and diet

We asked for information on dosage of NTBC at different age bands, number of doses per day and cumulative years of NTBC-treatment. Supply of amino acid mixtures and total protein supply were also subject of this part. Tyrosine and phenylalanine restriction and dietary compliance were specifically addressed.

### NTBC side effects

Participants were encouraged to report and specify any side effect caused by NTBC.

### Monitoring and follow-up

Centres supplied data on frequency and methods of liver and kidney imaging, ophthalmological, cardiac, neurological and skeletal examination. The frequency of outpatient-visits, developmental testing and dietitian visits per year were specified.

### Laboratory monitoring

We asked for data on the frequency of laboratory monitoring and range of target levels for tyrosine, alfa-fetoprotein, SA and delta- aminolevulinate.

Participants specified the frequency and target values of NTBC- levels (plasma/dried blood spots).

### Clinical outcome

Centres were asked for clinical sequelae and onset in each single patient.

If patients underwent liver transplantation, the participants were asked to supply data on indication, age, post-transplant tyrosine, SA and alfa-fetoprotein levels and complications.

### Statistical analysis

Statistical analysis was performed using SPSS version 20 and 21. Descriptive analysis of data was carried out. Analysis of age-dependency of symptoms and effects of early NTBC- treatment on outcome was performed using Pearson’s chi-squared test, if expected frequency was below 5 (20%) Fisher’s exact test was used. Odds ratio and confidence intervals were calculated. Normally distributed laboratory values were analyzed with ANOVA otherwise Kruskal-Wallis–test and Mann–Whitney-U-test were performed.

## Results

### Centres

21 centres sent data from individual patients. On average 7 patients (range: 1–20) were followed per centre. Dietitians were present in all centres, psychologists in 14/21 centres.

### Demographic data

168 patients with Tyr 1 from 21 centres were included. 100 patients were males and 68 females. Patients were aged between 0 and 24 years. The average follow-up period was 9.1+/− 6.3 years.

Consanguinity was reported in 49/168 cases and affected family members were found in 56/168 cases. 91 patients visited a mainstream school while 20/168 required special education.

### Diagnosis and clinical presentation

The average age at diagnosis was 12.9 months (SD: +/− 23.8, range: 0–183 months) for the whole group, 0.58 months (SD:+/−0.73, range 0–3 months) in patients diagnosed via NBS and 15.5 months (SD:+/−24.9, range 0–183 months) in those diagnosed via selective screening.

**Figure 2:**
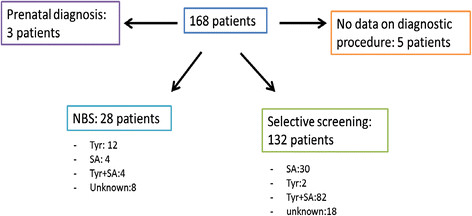
Distribution of patients according to diagnostic procedure.

Prenatal diagnosis was performed in 3/168 cases. 28/168 cases were diagnosed via NBS (Figure 2). 1/28 patient presented symptoms before or at diagnosis and suffered from renal dysfunction (nephromegaly, renal tubular dysfunction, nephrocalcinosis) at the age of 12 days. To our knowledge there were no false negative results when SA was used as a screening parameter. Only few patients diagnosed via NBS developed clinical symptoms after the start of therapy.

132 patients were diagnosed via selective screening. The screening parameter in these patients was SA (112/132 cases), mostly measured in urine. Data on initial symptoms were present in 118/ 132 patients. 4/118 children were selectively diagnosed because of an affected sibling and showed no clinical symptoms. Further details on diagnostic tools can be found in Figure 2.

114/168 patients developed symptoms. 40 patients had only one single symptom at diagnosis, mostly acute liver failure or liver dysfunction with bleeding tendencies and elevated liver enzymes (Table 1). 74 symptomatic patients showed a combination of symptoms, mainly liver dysfunction combined with renal dysfunction, in some cases combined with rickets. The remaining cases had unspecific symptoms such as fever, diarrhea, infections and feeding problems at diagnosis.

**Table 1:** Patients with a single symptom at diagnosis (100% = 40patients)

**Symptom**	**Patients (n)**	**%**
Acute liver failure	17	42.5
Liver dysfunction: bleeding tendencies, elevated liver enzymes	7	17.5
Hepatomegaly	2	5.0
Cirrhosis	5	12.5
Nephromegaly	1	2.5
Renal tubular dysfunction	1	2.5
Growth retardation	3	7.5
Rickets	1	2.5
Renal dysfunction	3	7.5

Mutation analysis was performed in 58/168 patients. The most frequent mutations were c.1062 + 5G > A (IVS12 + 5G > A) homozygous (11 patients) and c. 554-1G > T homozygous (13 patients). Using a retrospective study-design we could not find a genotype-phenotype correlation (both classifications of phenotype according to Halvorsen 1990 as well as according to van Spronsen 1994 were tested separately).

### Age-dependency of initial symptoms

**Figure 3:**
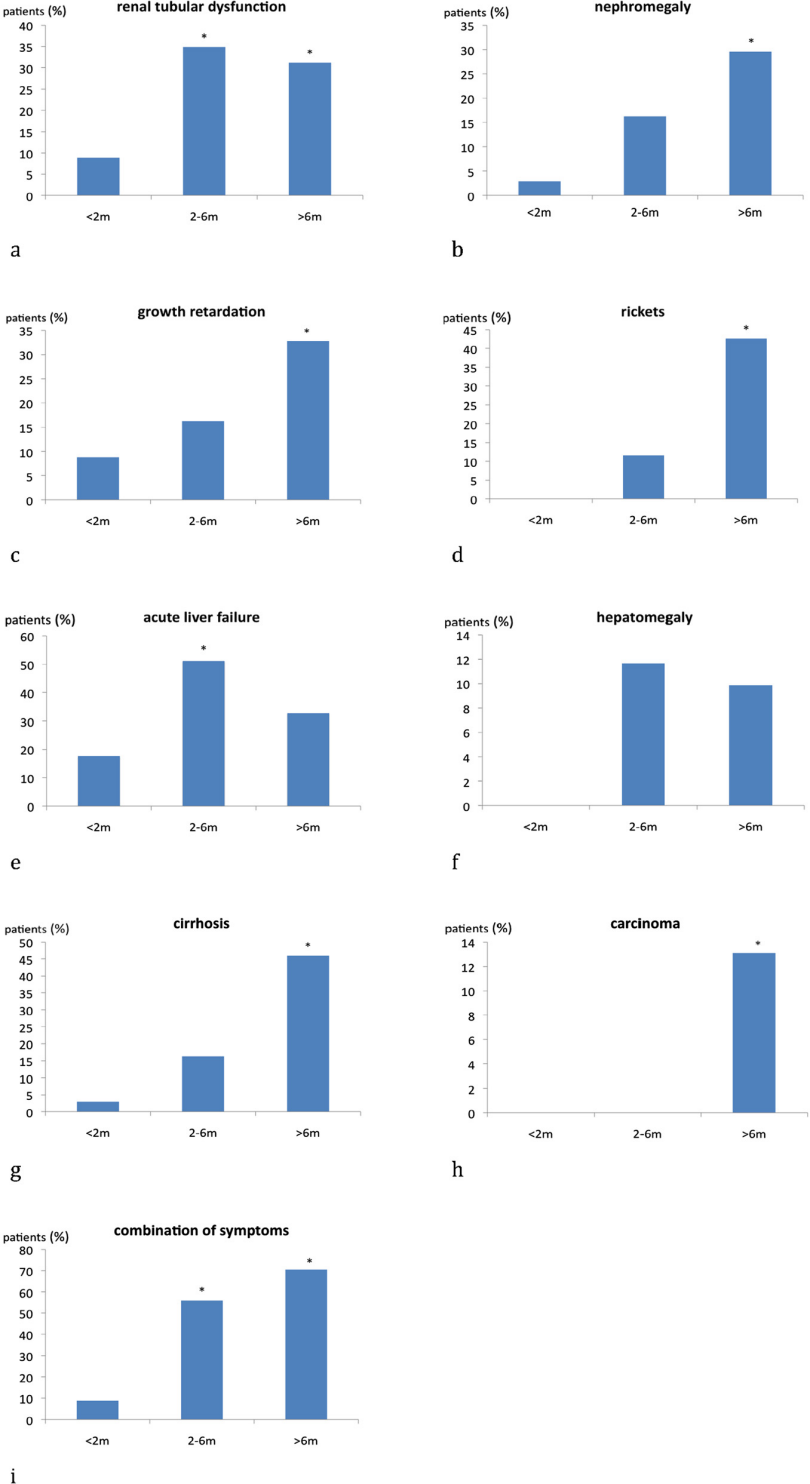
**Initial symptoms are age-dependent. a-i**: Age-dependency of initial symptoms: The most common symptoms like renal tubular dysfunction, nephromegaly, growth retardation, rickets, liver dysfunction and carcinoma and the combination of symptoms are depicted. *significant difference (p <0.05) vs < 2 months. n: <2 m =34 patients, 2-6 m = 43 patients, >6 m =61 patients.

Patients diagnosed via selective screening were less frequently symptomatic during the first months of life compared to older patients. Renal tubular dysfunction was more frequently found in patients between 2–6 months of age (35% affected; p = 0.007 vs < 2 months) and children beyond 6 months (31%; p = 0.013 vs < 2 months) compared to patients who were younger than 2 months (9%). Nephromegaly, rickets, growth retardation and liver cirrhosis were more often observed in patients beyond 6 months of age, who also developed more frequently HCC (13% affected; p = 0.047 vs < 2 months) compared to patients younger than 2 months at diagnosis (0%) (Figure 3b-f).

Hepatomegaly did not show any age-dependency. The number of patients suffering from acute liver failure was significantly higher in the group of patients between 2–6 months of age (51% affected; p = 0.002 vs < 2 months) compared to those who were younger (17%) or older (33%).

A combination of symptoms was found in 55% of patients aged between 2–6 months (p < 0.001 vs < 2 months) and in 70% of patients older than six months (p < 0.001), whereas a smaller number was observed in the group of patients below 2 months of age.

### Laboratory parameters at diagnosis and imaging

Parameters of liver function were generally normal in patients diagnosed via NBS or when prenatal diagnosis was performed, whereas liver dysfunction frequently occurred in patients detected by selective screening (Table 2).

**Table 2:** Initial laboratory findings of liver function (mean values)-a comparison between early screened and late diagnosed patients

	**≤ 6 months of age**	**> 6 months of age**	**Patients diagnosed via NBS**
**PTT**	80.5 seconds (SD: +/−40.6; median: 66.7; range: 39–181)	49.8 seconds (SD: +/−16.1; median: 51; range: 13.3-89.4)	84.4 seconds (SD: +/−39.1; median: 77.2; range: 45–168)
**AST**	113.3 U/l (SD: +/−116.7; median: 84.5; range: 29–613)	98.1 U/l (SD: +/−41.7; median: 88.5; range: 30–201)	45.5 U/l (SD: +/−21.9; median: 46; range: 18–72)
**ALT**	64.5 U/l (SD: +/−40.6; median: 59; range: 10–155)	59.6 U/l (SD: +/−30.2; median: 57.5; range: 16–124)	20.2 U/l (SD: +/−10.1; median: 21; range: 4–35)
**GGT**	158.2 U/l (SD: +/− 194.2; median: 97; range: (5–104)	158.2 U/l (SD: +/−108.7; median: 124; range: 29–515)	65.3 U/l (SD: +/−28.4; median: 66; range: 22–102)

The mean value of initial AFP in patients diagnosed via NBS was 277,107 μg/l (+/− SD: 200,449 μg/l, median: 318,309 μg/l; range: 20–587,000 μg/l). Patients < 6 months diagnosed via selective screening had initially a mean AFP value of 136,516 (+/− SD: 124,792 μg/l; median: 109,302 μg/l; range: 100–576,626 μg/l). Patients diagnosed beyond the age of 6 months, initially presented a mean AFP level of 41,487 μg/l ( +/− SD: 58,368 μg/l; median: 16,210 μg/l; range: 34–265,140 μg/l).

At diagnosis, initial liver imaging was performed in 79% of cases (110 selectively screened patients, 16 patients diagnosed via NBS, 3 patients with prenatal diagnosis, 3 patients with no data on diagnosis); all patients were examined by ultrasound of the liver. 19% of patients received a CT- and 24% MRI-scan in addition. Abnormalities in liver imaging were detected in 99/132 (75%) of patients, mostly nodules and cirrhosis (50%); 3% presented with carcinoma (age range 6–14 months); 6% of patients showed nephromegaly; in 21% increased echogenity was found. Only 4 patients diagnosed by newborn screening had abnormal liver imaging.

### Medication and diet

Data on NTBC treatment were obtained from 154/168 patients, 10/168 patients did not receive NTBC treatment; 6/10 patients were born before 1992 (i.e. before NTBC became routinely available), in 4/10 patients reasons are unknown. In 4/168 patients no data on treatment were available. Mean initial NTBC dosage was 1.7 mg/kg body weight (BW) per day (+/− SD: 0.5; range: 0.2-5 mg/kg BW per day). The number of cumulative years on NTBC treatment was 7.6 years +/− 5.1 years (range: 0–20 years) on average. In total 1157 NTBC years were observed. The dose decreased with age; during the first year of life the mean dose was 1.2 mg/kg BW per day (+/− SD: 0.3, range: 0.7-2); decreasing to 1.1 mg/kg BW per day (+/− SD: 0.3, range: 0.3-2.0 mg/kg BW per day) during the age interval 1–6 years. The dose at age 6–10 years showed a mean value of 1.0 mg/kg BW per day (+/− SD: 0.3, range: 0.05-2.6 mg/kg BW per day) and finally a slight decrease to 0.91 mg/kg BW per day (range: 0.2-2.6 mg/kg BW per day) after the age of ten years was found.

The current maintenance therapy was 1.0 mg/kg BW per day (SD +/− 0.3; range: 0.3-2). The daily dose was given in 1–3 doses per day, on average patients received 2 daily doses. NTBC-levels were monitored in 105 patients in plasma and in 46 patients in dried blood spots.

All patients tolerated NTBC without severe side effects. Some cases with transient side effects were reported (Table 3). Patients with side effects had NTBC levels between 32.3 to 71.1 μmol/l while NTBC levels in patients without side effects ranged from 31.3 to 58.4 μmol/l.

**Table 3:** Frequency of NTBC side effects (100% = 158 patients)

**NTBC side effect**	**Patients (n)**	**%**
Thrombocytopenia	8	5.0
Leukopenia	3	1.8
Eye pain	10	6.3
Eye itching	9	5.6
Conjunctivitis	3	1.8
Ceratitis	3	1.8
Corneal crystals	5	3.1
Epithelial abnormalities	1	0.63
Cognitive impairment	1	0.63
Behavioural disorder	1	0.63
Constipation	1	0.63
Myoclonia	1	0.63
Eczema	3	1.8

**Figure 4:**
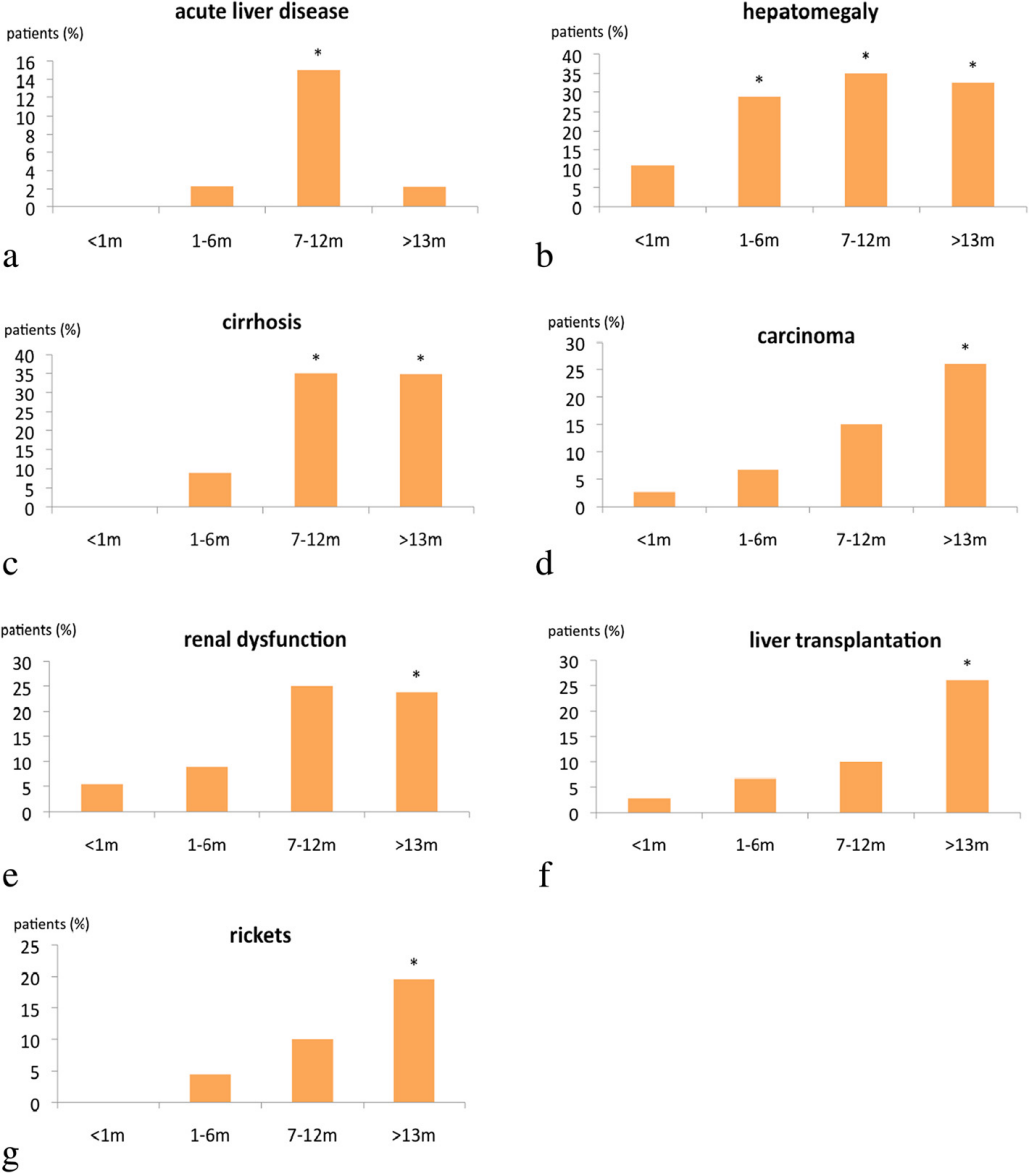
**The frequency of complications depends on the age at start of NTBC-treatment. a-g**: Age at initiation of NTBC treatment and frequency of complications: The most common complications like liver disease, HCC,renal dysfunction, rickets and necessity of liver transplantation are shown. *significant difference (p <0.05) vs < 1 month. n: < 1 m =37 patients; 1-6 m =45 patients; 7-12 m =20 patients; >13 m =46 patients.

Patients who were diagnosed and treated early (neonatal period) showed a lower rate of complications (Figure 4a-g), especially HCC (analysis was performed in 148 patients where age at NTBC start and clinical course were known). Delayed NTBC- treatment was associated with an increased risk of liver carcinoma and requirement of LTx. Treatment start within the age range of 1–6 months resulted in a 2.5-fold risk of developing liver tumors with requirement of LTx compared to patients with treatment start in the neonatal period. Patients aged between 7–12 months at start of NTBC treatment showed a 6-fold risk whereas children beyond 12 months of age had a 13-fold risk of tumor formation compared to the group of patients treated before the age of 1 month. Patients who received treatment beyond 6 months of age had a 5-fold risk of developing renal dysfunction. Table 4 summarizes the odds ratios for different clinical sequelae depending on the age at start of NTBC-treatment compared to start of treatment in the neonatal period. When NTBC treatment was initiated in the newborn period few clinical sequelae were observed: psychomotor impairment (7/28), ADHS and behavioral disorders (3/28), neurological crisis and learning difficulties (2/28), 1 patient suffered from convulsions which were unrelated to HT1 based on family history. Mild hepatomegaly (4/28), cirrhosis (1/28), nephromegaly (1/28), renal tubular dysfunction (1/28) were reported. Compliance with medication was not addressed in the questionnaire.

**Table 4:** **Age at NTBC-start and sequelae: odds ratio of patients diagnosed and treated beyond the perinatal period compared to those treated below the age of 1 month** ***(OR = Odds ratio; 95% LCI = lower limit of 95% confidence interval; UCI = upper limit of 95% confidence interval)***

**Complications**	**1-6 m** ***n=45 patients***			**7-12 m** ***n= 20 patients***			**>12 m** ***n= 46 patients***		
	**OR**	**LCI 95%**	**UCI 95%**	**OR**	**LCI 95%**	**UCI 95%**	**OR**	**LCI 95%**	**UCI 95%**
**LTx**	**2.5**	0.2	25.8	**4**	0.3	47.1	**12.7**	1.5	103
**acute liver failure**	**2.5**	0.1	63.9	**15**	0.7	306.4	**2.4**	0.09	62.4
**Carcinoma**	**2.5**	0.2	25.8	**6.3**	0.6	65.6	**12.7**	1.5	103
**Cirrhosis**	**8.1**	0.4	156.1	**41.6**	2.2	779.9	**40.5**	2.3	704.1
**hepatomegaly**	**3.3**	0.9	11.3	**4.4**	1.1	17.7	**3.9**	1.1	13.3
**Rickets**	**4.3**	0.2	92.6	**10.1**	0.5	222.1	**19**	1.1	338.3
**renal dysfunction**	**1.7**	0.2	9.8	**5.8**	1.0	33.4	**5.5**	1.1	26.6
**renal tubular dysfunction**	**1.2**	0.1	7.9	**1.9**	0.2	14.9	**4.3**	0.8	21.6
**nephromegaly**	**0.8**	0.1	6	**3.0**	0.4	20.2	**2.6**	0.4	13.8
**nephrocalcinosis**	**4.3**	0.2	92.6	**5.7**	0.2	148.3	**2.5**	0.09	62.5
**impaired growth**	**2,5**	0.1	63.9	**1.8**	0.03	95.6	**2.5**	0.09	62.5
**Adipositas**	**2.5**	0.1	63.9	**1.8**	0.03	95.6	**2.5**	0.09	62.5
**neurological concomitant disease**	**1.4**	0.3	6.3	**0.5**	0.05	6.1	**0.2**	0.02	2.5
**neurological crises**	**0.2**	0.02	2.5	**0.5**	0.05	6.1	**0.2**	0.02	2.5
**Epilepsy/convulsion**	**0.8**	0.1	3.4	**0.9**	0.1	5.5	**0.1**	0.02	1.7
**ADS, behavioural disorders**	**1.4**	0.3	6.3	**1.2**	0.1	8.2	**0.5**	0.08	3.2
**learning/language difficulties**	**0.8**	0.05	13.5	**0.5**	0.02	15.2	**0.2**	0.01	6.6
**Impaired psychomotor development**	**0.7**	0.2	2.3	**1.5**	0.4	5.34	**0.7**	0.2	2.27
**Death**	**0.8**	0.01	42.5	**15**	0.7	306.4	**2.5**	0.09	62.5

**Figure 5:**
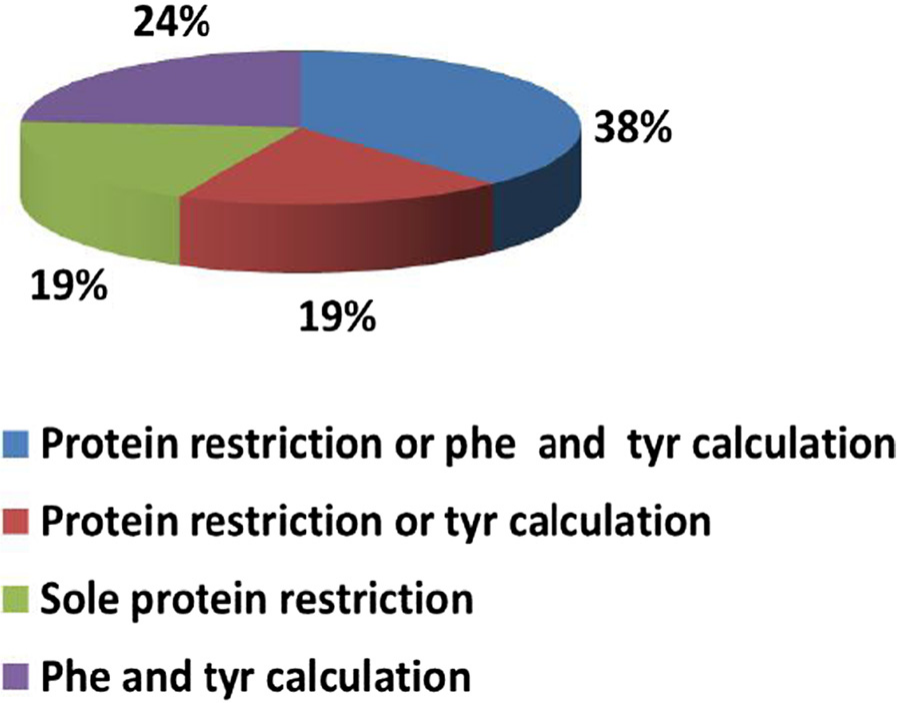
**Differences in dietary treatment.** Phe = Phenylalanine; Tyr = Tyrosine.

In addition to NTBC administration, all patients received dietary treatment. 16 centres added micronutrients to the diet and supplemented vitamins. 38% of centres treated their patients either with natural protein restriction or calculated phenylalanine and tyrosine intake. 19% of centres restricted either natural protein intake or calculated tyrosine intake. 19% treated their patients with natural protein restriction only and 24% of centres stated to exclusively use restriction of phenylalanine and tyrosine (Figure 5). Tyrosine restriction/calculation was done in some centres in younger patients during the first year of life whereas diet was more relaxed in older patients.

The total protein intake decreased with age. Patients aged between 0–12 months at diagnosis had an initial natural protein intake of 1.4 g/kg per day (+/− SD: 0.79) supplemented with 1.2 g/kg per day via amino acid mixture. Further values can be found in Table 5.

**Table 5:** Protein intake and age bands (g/kgBW per day)

**Age bands**	**Total protein intake (mean +/−SD)**	**Intake via amino acid mixture (mean +/−SD)**
**0-1 y**	1.4 +/− 0.5	1.1 +/− 0.1
**2-5 y**	1.5 +/− 0.5	0.9 +/− 0.4
**6-10 y**	1.0 +/− 0.6	0.8 +/− 0.36
**11-15 y**	0.9 +/− 0.6	0.7 +/− 0.37
**16-20 y**	0.6 +/− 0.3	0.7 +/− 0.37
**>20 y**	0.5 +/− 0.2	0.8 +/− 0.15

### Liver transplantation

29/168 patients underwent liver transplantation; main indication was hepatocellular carcinoma (72%), liver cirrhosis and liver failure were other sequelae necessitating liver transplantation. The mean age was 80.5 months (+/− SD: 64.5; range:7–252).

Four patients showed posttransplant morbidity, but did not have to undergo retransplantation. Five cases (21%) suffered from complications after liver transplantation and underwent re-transplantation due to failure of the transplanted liver. 2/5 patients died after retransplantation; 3/5 died despite re-retransplantation.

Post-transplant tyrosine levels were: mean 198 μmol/l (range: 17–756 μmol/l; normal: >1 month of age 28–153 μmol/l); AFP levels: mean 6.7 μg/l (range: 0.9-27.7 μg/l, normal: <7 μg/l). After transplantation, additional NTBC- treatment was given to 6 patients from 3 different centres. 4 of them received a restriction of total protein.

### Clinical sequelae

The most frequent complications in NTBC treated patients during the clinical course were impaired psychomotor development, hepatomegaly and cirrhosis (Table 6). Acute liver failure seems to be a very rare complication (5/148 patients) in late diagnosed and treated patients (>6 months of age). Clinical sequelae were rarely observed in patients treated early (<1 month of age) with NTBC (Figure 4). The rate of clinical complications critically depends on the age at start of treatment (Table 4). 1/148 treated patient died because of failure of the transplanted liver; the patient was diagnosed and treated at the age of 5 years. 2/148 patients (at diagnosis: 8 months and 10 months of age) died from HCC (metastasis).

**Table 6:** Clinical outcome and frequency of different symptoms (mean age at onset and age range) in the treated cohort (100% = 148 patients)

**Outcome**	**Patients**	**%**
**Liver transplantation**	18	12.2
**Acute liver disease***(12 months; SD: 9.2; range: 5–28)*	5	3.4
**Carcinoma***(74.3 months; SD: 67; range: 6–216)*	19	12.8
**Cirrhosis***(19.2 months; SD: 16.4; range: 3–72)*	27	18.2
**Hepatomegaly***(26.2 months; SD: 16.4; range: 1–204)*	29	19.6
**Portal hypertension**	1	0.7
**Renal dysfunction (in general)**	22	14.9
**Renal tubular dysfunction ***(28.4 months; SD: 32.2; range: 4–114)*	16	10.8
**Nephromegaly***(23.3 months; SD: 20.6; range: 8–68)*	13	8.8
**Nephrocalcinosis***(30 months; SD: 33.9; range: 6–54)*	4	2.7
**Nephrolithiasis, compensated renal failure**	1	0.7
**Rickets***(28.3 months; SD: 21.4; range: 5–54)*	13	8.8
**Impaired growth**	2	1.4
**Adipositas**	2	1.4
**Cardiomyopathy***(28 months)*	1	0.7
**Neurological concomitant diseases***(55 months; SD: 40.9; range: 7.1-96)*	10	6.8
**Neurological crisis***(41.2 months; SD: 23.2; range: 9–60)*	6	4.1
**Epilepsy and convulsion**	11	7.4
**Hyperactivity, ADHS, behavioural disorders**	12	8.1
**Learning/language difficulties, dyslexia**	2	1.4
**Impaired psychomotor development***(29.4 months; SD: 29.3; range: 3–96)*	30	20.3
**Death**	3	2.0

### Therapeutic monitoring

The amino acid profile was monitored by 95% of centres. 10% of centres used urine and 48% dried blood spots, 42% plasma. The maximal acceptable plasma level of tyrosine varied between 200–800 μmol/l (mean: 481.7 +/− 118 μmol/l).

SA was measured by all centres. Monitoring of SA was performed in blood by 29% of centres, by 81% of centres in urine and 62% of centres used dried blood spots. 86% of centres stated that the target of SA should be below detection limit. 14% of centres gave a target value for SA levels in plasma below 0.1 μmol/l and in urine below 0.1 mmol/mol creatinine.

Urinary delta-aminolevulinate and metabolites were measured in 10/21 centres.

Monitoring of NTBC plasma levels was performed in 76% of centres; 38% used dried blood spots; 76% of centres measured NTBC in serum. 33% of centres used standardized sampling with trough levels of 30–50 μmol/l (7 centres) and peak levels of 50–100 μmol/l (6 centres) and 29% preferred random sampling with target levels between 20–80 μmol/l.

### Follow-up

All centres performed regular blood count. 43% of centres assessed vitamin A and vitamin E, 57% of centres measured vitamin D and vitamin B12 in their patients. Folate was measured by 48% of centres and micronutrients by 62% of centres.

Biochemical parameters like methylmalonic acid in urine or in blood, homocysteine, IGF1, selenium, zinc, TSH, parathyroid hormone, ferritin, CRP, bilirubin, electrolytes, tubular function, carnitine and prealbumin were not regularly determined in all centres.

### Liver assessment

PTT was measured by 91% of centres; PT by 76%. AST/ALT levels were monitored by 76% of centres whereas gamma GT was measured by 95% of centres. 90% of centres included measurement of albumin in their routine procedures and 95% of centres stated to measure alkaline phosphatase. All centres monitored AFP levels and regularly performed ultrasound of the liver. 76% of centres stated to perform MRI in addition. 5% of centres used CT for liver imaging. The frequency of liver imaging varied from every 3 months to once a year. 62% recommended yearly liver imaging.

### Renal evaluation

GFR and cystatin c were assessed by 5% of centres. Serum urea, calcium and phosphate were measured by 95% of centres. All centres stated to measure serum creatinine. Renal ultrasound was performed by 85% of centres. 67% of centres indicated to carry out renal imaging once a year. Some centres performed renal imaging every 3 months or every 6 months. In 1 centre MRI of kidneys was regularly carried out.

### Psychomotor assessment

Assessment was performed by 14 out of 21 centres (65%), frequencies differed from once per year, every two or three years to assessment according to individual needs or age. Most centres performed evaluation once a year.

### Neurological follow-up

Follow-up was done in all centres by clinical examination. The frequency varied from at least once a year to maximal every 3 months. EEG was performed on a regular basis by 1/21 centres.

### Cardiac follow-up

Cardiac follow-up was carried out by 14/21 centres.

### Skeletal examination

12 out of 21 centres (57%) performed skeletal examination. 8 centres used x-ray in their patients and 8 centres performed osteodensitometry.

### Ophthalmological examination

Follow-up via slit lamp examination was done by 15/21 centres. In 11/15 centres (71%) a yearly examination took place.

Other centers recommended eye examination every 6 months, twice a year, once after diagnosis or every 2 years, some order eye examination only when clinical symptoms occur.

### Refund of expenses

In almost all centres (20/21) patients received refund of expenses for NTBC and tyrosine –free amino acid mixture and in 10/21 centres patients obtained reimbursement for protein-restricted diet.

## Discussion

Data from 168 patients with Tyr 1 were included in our study. This may seem a small number, however the total number of patients treated with NTBC in Europe is less than 400 (personal communication, Erik Brouwer, Swedish Orphan Biovitrum). We observed considerable differences among centres in Europe, Turkey and Israel regarding diagnosis, treatment and monitoring of Tyr 1.

### Diagnosis

In our study, patients who were diagnosed after the neonatal period and consequently received NTBC-treatment later, had a 2-12-fold higher risk (depending on age at start of therapy) of developing hepatocellular carcinoma compared to patients treated as neonates. This is in line with results from earlier smaller studies showing an increased risk for hepatocellular carcinoma when NTBC treatment was initiated at a later age [12],[15],[21]. Our results show that the risk for HCC is already increased when treatment is initiated beyond the newborn period.

As patients were often asymptomatic in the first few months of life and the initial clinical symptoms were rather unspecific newborn screening is mandatory to diagnose patients early in life. Tyrosine levels in blood (spots) are an inappropriate screening parameter lacking both sensitivity and specificity [22]. In contrast, SA in dried blood is a specific and sensitive screening parameter which can be indirectly [23] or directly [22],[24]-[29] determined, additional measurement of tyrosine in dried blood is optional.

Despite compelling evidence that NBS can prevent liver carcinoma, still 43% of the participating centres do not screen for Tyr 1. The majority of patients in our study (132/168) were diagnosed by selective screening after presentation of clinical symptoms.

Higher initial AFP levels in patients screened via NBS compared to those who were diagnosed late are partly due to age-dependency of AFP levels [30],[31].

In line with previous studies, we could not find a clear genotype-phenotype correlation [32] using two classifications described by Halvorsen 1990 and van Spronsen 1994, respectively [33],[34]. Some mutations are population-specific [35], nevertheless similar to Couce et al. [13] we found IVS 6 -1(G > T) to be one of the most common mutations, but IVS12 + 5G > A (c.1062 + 5G > A) was frequent as well. Lack of genotype-phenotype correlation may partly be due to genetic reversion, as mosaicisms in liver nodules can be found and self –induced genetic corrections in liver tissue may improve the clinical phenotype [36],[37].

### Implications for clinical practice

A delay in instituting NTBC-treatment beyond the neonatal period results in an increased risk for HCC (up to 12 fold in children older than 12 months). When SA is used as a screening parameter, NBS for Tyr 1 fulfils the criteria laid down by Wilson and Jungner [38]. In most patients, there is latency until (irreversible) clinical symptoms occur, a specific and sensitive screening test exists, efficient therapy is available and the test is acceptable for the public. Our results underpin the importance and necessity of newborn screening using SA as a screening parameter, tyrosine may be used as an additional parameter.

Mutation analysis is not essential for clinical management but is useful for prenatal diagnosis and reproductive counselling.

### Treatment and Monitoring of Therapy

The starting dose was 1–2 mg/kg BW per day in all centres. In younger patients with liver failure, a loading dose of 2 mg/kg BW per day for 1 week was given. The initial dose could be titrated down in most patients (even as low as 0.3 mg/kg per day) without risking metabolic control as judged by SA levels/excretion [39]. But it has to be pointed out that levels of NTBC may fluctuate during intercurrent illness (e.g. fever, infections), therefore very low levels should be avoided.

NTBC-treated FAH −/− knock-out mice developed liver failure or died after complete withdrawal of NTBC [40]. In this animal model, discontinuation and restart of NTBC treatment favoured formation of liver tumours while continuous low-dose NTBC treatment promoted regeneration of liver lesions in these FAH−/− knock-out mice (personal communication AV and JE). Whether these findings can be transferred to clinical medicine is not clear. Additionally, patients may develop neurological crises in response to discontinuation of NTBC [41]. As shown in Table 6 some (transient) symptoms (mainly liver and kidney) may occur in the early phase after treatment initiation.

In our survey, 2 daily doses were given on average. Due to the long half-life of NTBC, once daily dosing may be an option in older children to increase compliance. Pharmacokinetics of NTBC in smaller children is still unclear. The publication by Schlune et al. [42] advocating once daily dosing sheds some indirect light on the pharmacokinetics of NTBC in children. Typical side effects of NTBC were transient thrombocytopenia, leukopenia and ocular symptoms which are comparable to data from the post marketing survey (personal communication, Erik Brouwer, Swedish Orphan Biovitrum). In our data collection, only 8 patients had thrombocytopenia and 3 patients developed leukopenia. On average, patients with side effects seem to have a higher range of NTBC values compared to those with no side effects, however the sample size of patients with side effects was small; a statistical analysis was not possible. Transient ocular symptoms were reported in patients with poor dietary control. It was previously reported that ocular symptoms were associated with high tyrosine levels [43], whereas in another study patients with high plasma tyrosine levels did not develop any ocular symptoms [44].

### In all centres, metabolic control was monitored using SA concentration in dried blood and/or urine, the target level is below detection limit

Not all centres monitored NTBC–levels in patients. While metabolic control can be judged by SA levels/excretion in urine overdosing and compliance can only be judged by measuring NTBC-levels. Monitoring of NTBC plasma levels is therefore useful and permits individual dosing. Thus, treatment costs and side effects can be minimized without hampering metabolic control. The target level of NTBC varied among centres. Target levels are not well defined, in the literature, different target levels of NTBC have been suggested [39],[42],[45]-[47].

The centres unanimously used dietary treatment in Tyr 1. However, centres had different approaches concerning dietary treatment and supplementation of micronutrients and vitamins. Maximal acceptable plasma tyrosine levels in the different centres ranged from 200–800 μM. As some centres used plasma, while others used dried blood this broad range may be partly due to using different sampling material; Concentrations measured in dried blood are about 1.4 times lower compared to plasma [39], unless normalized to haemoglobin content [48]. Recently, diurnal variation of phenylalanine in Tyr I patients has been detected [49], the clinical significance for the outcome is unknown. In our survey, frequency of tyrosine measurements depended on age and biochemical stability.

### Implications for clinical practice

Tyrosine restriction is recommended. It is not clear whether protein restriction or calculation of tyrosine and phenylalanine intake should be preferred. Judged by clinical experience, we think that tyrosine and phenylalanine calculation is justified in younger children during their first 1–2 years of life and not necessary in older patients. Regular intake of a tyrosine-free amino acid mixture divided in 3 daily doses is essential to improve uptake of amino acids. Micronutrients, vitamins and phenylalanine should be supplied according to blood levels and/or dietary protocols.

Based on discussion and experience, the workshop group agreed on a target tyrosine level in plasma below 400 μM which is regarded as both safe and feasible. This was also recommended by other groups [2],[18].

Continuous NTBC-treatment at a starting dose of 1–2 mg/kg per day is necessary to avoid sequelae. We recommend adjusting the individual NTBC dose according to NTBC- and SA- levels in order to minimize side effects and costs. Based on a review of the literature and personal experience, the workshop group recommends a NTBC plasma level between 20 and 40 μM at random sampling. Once daily dosing may be an option in older children to improve compliance. In younger children pharmacokinetics of NTBC are not yet clear twice daily dosing seems to be adequate in this group. Random sampling is possible as NTBC has a long half-life of 54 h [50]. NTBC levels in dried blood spots are 1.4-1.6 times lower compared to plasma [39] unless samples are normalized to haemoglobin content [48].

Prospective data related to outcome are necessary to underpin our opinions regarding target values for tyrosine and NTBC as well as a correlation between NTBC levels and side effects.

### Liver transplantation

In our study, liver transplantation was the therapy of choice in patients with hepatocellular carcinoma. Further indications were failure to respond to NTBC (therapy-resistant liver failure with insufficient improvement despite treatment with a NTBC-loading dose of 2 mg/kg per day for one week), chronic liver disease or poor quality of life due to dietary restriction and frequency of blood sampling which is comparable to previous findings [51].

Stabilization by NTBC-treatment before liver transplantation improved the outcome. Patients with a high post-transplant tyrosine plasma level or urinary SA excretion may benefit from protein restriction and low-dose NTBC treatment [52],[53]. In our retrospective study, 6 patients from 3 different centres received NTBC and 4 of them were treated with a protein restricted diet after transplantation with a good outcome.

### Implications for clinical practice

Liver transplantation is indicated in hepatocellular carcinoma and NTBC-resistance. Whether post-transplant NTBC and dietary treatment in patients excreting SA in urine after transplantation is necessary should be examined in future studies.

### Monitoring and follow-up

The frequency of follow-up procedures, techniques and scope of laboratory examinations were heterogeneous among centres. Comparable heterogeneities of follow-up procedures have been reported by a French–Belgian study group in a local study [16].

Dried blood spots are convenient for home monitoring, especially useful in remote areas for simultaneous measurement of succinylacetone, tyrosine, phenylalanine and NTBC [39],[48].

Ultrasound of the liver was regularly performed, the frequency of imaging varied from every 3 months to once a year. Ultrasound of the liver in experienced hands seems to be appropriate for follow-up. MRI was performed in most centres when malignancy was suspected. Occasionally, nodules are present before initiation of NTBC-treatment but regress on repeated imaging following treatment [18].

In our study, Fanconi-syndrome-like tubulopathy and hypophosphataemic rickets were the most common renal manifestations of Tyr 1. The tubulopathy associated with Tyr 1 was previously reported to be reversible [54], however progressive tubular dysfunction can lead to glomerular dysfunction [53]. According to the group’s experience, urinary testing should be performed once a year or until normal values are obtained using protein, glucose, beta1-albumin, alfa1-microglobuline and albumin.

In our case-series, minor cardiac dysfunction was only observed in one single patient who presented with minor cardiomyopathy. Cardiomyopathy in treated Tyr 1 is a rare complication mostly with a favourable outcome [55]-[57], cardiac tests are only necessary when clinical symptoms occur.

Half of the centres performed skeletal examination (x-ray or osteodensitometry). In our study, bone symptoms like rickets were rare, corneal crystals with clinical symptoms were only found in 8 patients. Hence skeletal and ophthalmologic tests are only necessary when clinical symptoms exist.

Well-known neurological features of Tyr 1 are porphyria-like crises and peripheral neuropathy. These were rare in our cohort. Only recently, cognitive deficits were described in some Tyr 1-patients treated with NTBC [5]-[8],[11]. In our study, 20% of patients had psychomotor impairment and 17 patients (11.5%) required special education. However, regular psychomotor and intelligence testing is rarely performed (14/21 centres). More widespread testing is warranted in order to better characterize these problems and offer specific support. The mechanisms leading to psychomotor impairment are still unclear. Elevated levels of tyrosine, other toxic metabolites and NTBC have been discussed to play a role. Tyrosine may compete with the transport of other amino acids to the brain. The synthesis of neurotransmitters might be altered by NTBC-related hypertyrosinaemia [5].

### Implications for clinical practice

The workshop group recommends ultrasound of the liver every 6 months and monitoring of AFP levels every 3–6 months. Contrast-enhanced ultrasound is more sensitive. When malignancy is suspected, MRI of the liver with additional measurement of AFP blood levels is recommended as has been suggested before [58]. Based on clinical experience, the group judges MRI to be more sensitive in detecting tumours compared to ultrasound of the liver.

Regular monitoring of tubular function is recommended.

Cardiac follow-up, osteodensitometry and slit lamp examination of the eyes is not considered obligatory but should be performed according to clinical and biochemical pathology.

A regular psychomotor assessment is highly recommended in order to support affected children by special education. Further studies are necessary to elucidate the underlying pathophysiology.

### Unmet needs

Financial support is an important issue for access to an optimal treatment; in almost all centres, patients received refund of expenses for NTBC and tyrosine-free amino acid mixture. But only patients in half of the participating centres obtained reimbursement for a protein-restricted diet which might affect the access and adherence to treatment.

### Implications for clinical practice

Costs for neonatal screening and treatment have to be born by the government or health insurance companies to allow early diagnosis and access to adequate treatment.

## Conclusions

We present data from a retrospective, cross sectional study including a substantial number of 168 patients with Tyr 1.

Our results underpin the urgent need for NBS in Tyr 1 using SA as a screening parameter. The risk for developing HCC in the untreated patient increases during the first months of life by a factor of 13 compared to newborns. Treatment consists of a protein reduced diet and NTBC. Once daily dosing may be an option in older children to increase compliance. NTBC should be individually dosed to minimize both side effects and costs. Target levels suggested by our group are 20–40 μM for NTBC and 400 μM for tyrosine in plasma. Further prospective studies including outcome parameters are needed for confirmation.

For monitoring liver carcinoma, our group judged (contrast-enhanced) ultrasound in combination with tumour markers appropriate as a routine procedure, MRI should only be performed if malignancy is expected. Hepatic and renal function has to be assessed regularly. Evaluation of bone, ocular and cardiac function is only recommended in the presence of clinical symptoms. Neurocognitive deficits have been frequently observed in our cohort. It is not clear whether these are symptoms of the disease or side-effects of medication. Regular assessment of psychomotor and cognitive function is recommended. Prospective studies are required to further characterise neurocognitive outcome in Tyr 1.

Clinical implications deducted from our findings and unsolved issues arising from our study and workshop discussion are summarized in Table 7.

**Table 7:** Summary of clinical practice recommendations and issues to be addressed in future (prospective) studies

	**Clinical practice recommendation**	**Issues to be addressed in future studies**
**Diagnosis**	•NBS via SA to avoid sequelae including HCC	
**Treatment & Monitoring**	•NTBC & diet	•Calculation Tyr & Phe vs protein restriction
	•NTBC-level 20-40 μM	•NTBC-target level
	•Tyr-level < 400 μM	•Tyr-target level
	•SA below detection limit	•SA target level
	•Contrast enhanced ultrasound & AFP	•Correlation NTB-levels & side effects
	•Liver & Kidney function	•NTBC- pharmacokinetics in children
**Liver transplantation**	•HCC	•NTBC & diet if SA-elevation post transplant
	•Therapy resistance	
**Outcome**	•Psychomotor/neurocognitive assessment	•Pathophysiology of neurological damage and risk factors
		•Definition of adequate test battery
**Reimbursement**	•Diagnosis & Treatment & Monitoring	

## Abbreviations

AFP: Alfa-fetoprotein

CEA: Carcinoembryonic antigen

CT: Computed tomography

FAH: **−/−** Fumarylacetoacetate gene knock-out

HCC: Hepatocellular carcinoma

LTx: Liver transplantation

MRI: Magnetic resonance imaging

NBS: Newborn mass screening

NTBC: [2-(2-Nitro-4-trifluoromethylbenzoyl)-1, 3-cyclohexanedione; nitisinone]

SA: Succinylacetone

Tyr 1: Tyrosinaemia type 1

## Competing interest

The authors declare that they have no competing interests.

## Authors’ contributions

SM participated in the workshop and coordination of the study, collected data, carried out the statistical analysis and drafted the manuscript. SE participated in the design of the study and supported the statistical analysis. UM, JE, JJ, AV, PM, SM, JS participated in the workshop and contributed to the manuscript and discussion with their expertise. GG, NGS, HODB, FJVS, JZ, CDL, US, ET, AM, CDV, DM, MB-K, ASL-H, JC, MLC, RS, SS-B, HM, YTB, PF, L A-E, MH, MG participated in the workshop and contributed patient data and ideas for analysis. AMD planned and supervised the study, participated in the workshop and finalized the manuscript. All authors read and approved the final version of the manuscript.
